# Supplementary material for: Nonlinear fusion is optimal for a wide class of multisensory tasks
Source: PLoS Comput Biol. 2024 Jul 5;20(7):e1012246. doi: 10.1371/journal.pcbi.1012246 (PMC11253934; doi:10.1371/journal.pcbi.1012246)
Supplement: S1 File — (PDF) [file pcbi.1012246.s001.pdf]

# Supporting information: Nonlinear fusion is optimal for a wide class of multisensory tasks

**Marcus Ghosh**
Gabriel Béna
Volker Bormuth
Dan F. M. Goodman

## A. In linear models, early and late fusion are equivalent

If $x_{ct}$ is the evidence in channel $c$ at time $t$ then early fusion can be represented by the equation $\sum_t f(\sum_c x_{ct})$ for some fusion function $f$, while late fusion would be represented as $\sum_c f(\sum_t x_{ct})$. If the function $f$ is linear then these two are equivalent:

$$
\begin{aligned}
& \textstyle\sum_t f(\sum_c x_{ct}) && \text{(Early fusion)} && (17)\\
= \;& f(\textstyle\sum_t \sum_c x_{ct}) && \text{(by linearity of } f) && (18)\\
= \;& f(\textstyle\sum_c \sum_t x_{ct}) && \text{(exchanging summations)} && (19)\\
= \;& \textstyle\sum_c f(\sum_t x_{ct}) && \text{(Late fusion, by linearity of } f) && (20)
\end{aligned}
$$

## B. Reliability is not well defined for non-independent channels

We do not know of any attempt to precisely define the reliability of a channel in a multimodal setting. However, let us assume that any such definition would have the property that if all channels had zero reliability, then it would not be possible to solve the task. We can show that this property cannot hold in general when channels are not independent, and therefore there cannot be a sensible definition of reliability. For example, consider the following two channel task definition:

$$
\begin{aligned}
M &= \pm 1 \text{ with equal likelihood} && (21)\\
C_{0t} &= \pm 1 \text{ with equal likelihood} && (22)\\
C_{1t} &= C_{0t} M && (23)
\end{aligned}
$$

Clearly, $C_{0t}$ is independent of $M$ so just knowing $C_{0t}$ tells you nothing about $M$ and therefore channel 0 has zero reliability. Similarly, if you only know $C_{1t}$ then this indicates nothing about the value of $M$ because you do not know if it is $M$ or $-M$ at any given time $t$. Therefore, channel 1 also has zero reliability. However, simply computing $D_t = C_{0t}C_{1t} = C_{0t}^2 M = M$ immediately tells you $M$ even from a single observation.

## C. Figures

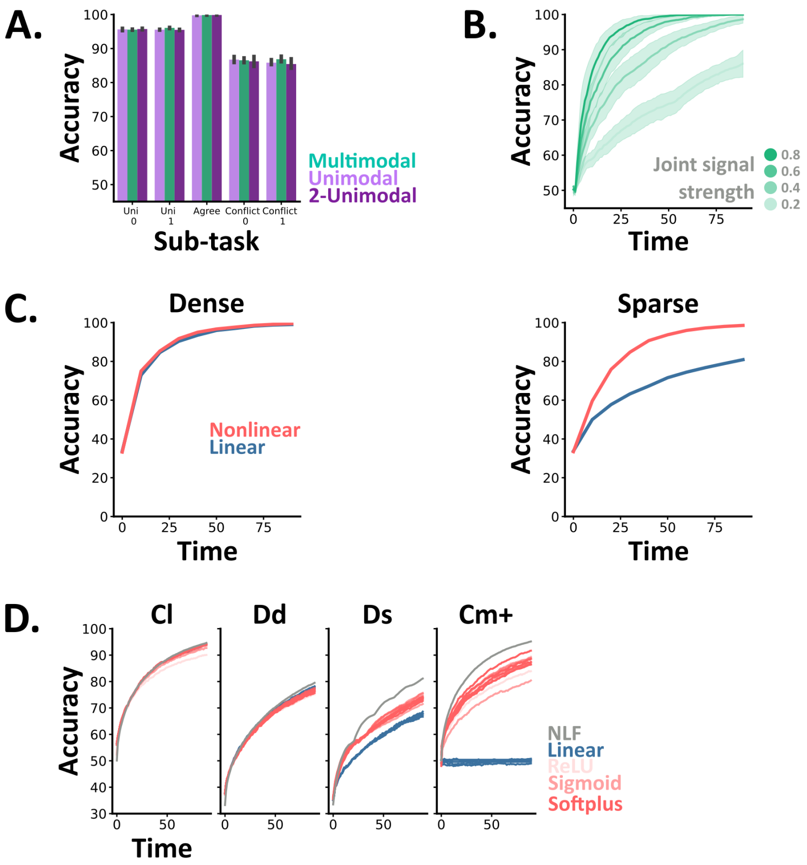


**A.** Each architecture's (colours) test accuracy (mean and std across networks) per subtask in the extended classical task.

**B.** The accuracy over time (the number of elapsed time steps within a trial) of multimodal networks trained on the comodulation task with multiple joint signal strengths. Each line shows the mean and std (shaded surround) across networks, and trials with different joint signal strengths (darker green = higher).

**C.** Accuracy over time curves for linear (blue) and nonlinear (coral) fusion in dense (left) and sparse (right) settings with 5 channels and continuous, rather than discrete, directions.

**D.** Accuracy over time curves for ANN models with different multisensory activation functions (colours), trained and tested on different tasks (subplots). Ideal nonlinear fusion accuracy is overlaid in grey. Tasks, Cl: reduced classical, Dd: dense detection, Ds: sparse detection, Cm+: probabilistic comodulation.

E.

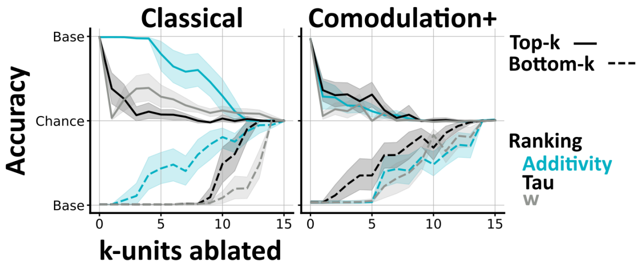


F.

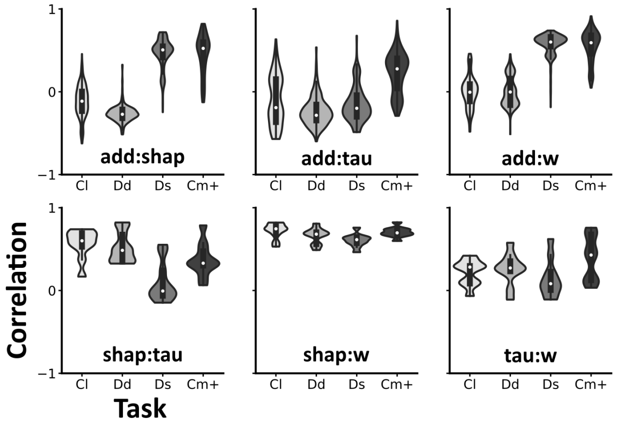


**E.** Network accuracy, from baseline to chance, as we ablate either the top (solid lines) or bottom (dashed lines) k-units, ranked by different unit properties (colours). We plot the mean and std across networks, and invert the y-axis for the bottom-k results.

**F.** Correlations between different unit properties (add: additivity, tau: membrane time constant, $w$: mean input weights) and their causal contribution to task performance (shap: Shapley value). Tasks, Cl: reduced classical, Dd: dense detection, Ds: sparse detection, Cm+: probabilistic comodulation.

## D. Task code

Below, we provide sample Python code to make clear how we generate trials in each task. In our working code, we actually specify probability distributions using the Lea library [1], and used this both to efficiently generate samples and compute the log odds for the maximum a posteriori estimators. This code is included in the repository, but is not shown here as it requires familiarity with the Lea library.

### Classical task

```
from numpy.random import choice
def classical_trial(n, s):
    M = choice([ -1, 1], p=[0.5, 0.5])
    p = [(1 -s)/3, (1 -s)/3, (1+2*s)/3]
    A, V = choice([ -M, 0, M], p=p, size=(2, n))
    return M, A, V
```

### Extended classical task

```
from numpy.random import choice
def classical_trial(n, s_A, s_V):
    M_A = choice([ -1, 1], p=[0.5, 0.5])
    M_V = choice([ -1, 1], p=[0.5, 0.5])
    p = 1.0*(s_A>s_V)+0.5*(s_A==s_V)
    M = choice([M_A, M_V], p=[p, 1 -p])
    p_A = [(1 -s_A)/3, (1 -s_A)/3, (1+2*s_A)/3]
    p_V = [(1 -s_V)/3, (1 -s_V)/3, (1+2*s_V)/3]
    if s_A== -1:
      p_A = [0, 1, 0]
    if s_V== -1:
      p_V = [0, 1, 0]
    A = []; V = []
    for t in range(n):
        A.append(choice([ -M, 0, M], p=p_A))
        V.append(choice([ -M, 0, M], p=p_V))
    return M, A, V
```

### Generalised comodulation task

```
from numpy.random import choice
def comod_trial(n, pcc, pii, pci, pcn, pin, pnn):
    M = choice([ -1, 1], p=[0.5, 0.5])
    # dictionary of probabilities of particular pairs (A, V)
    dist = {(M, M): pcc, ( -M, -M): pii,
            (M, -M): pci, ( -M, M): pci,
            (M, 0): pcn, (0, M): pcn,
            ( -M, 0): pin, (0, -M): pin,
            (0, 0): pnn}
    # flat list of pairs and probabilities
    av_pairs = list(dist.keys())
    p = list(dist.values())
    # generate sequence of observations
    A = []; V= []
    for t in range(n):
        pair_index = choice(len(p), p=p)
        a, v = av_pairs[pair_index]
        A.append(a)
        V.append(v)
    return M, A, V
```

### Perfectly balanced comodulation task

```
from numpy.random import shuffle, choice
# n must be a multiple of 3, to ensure exact repartitions of directions
def perfect_comod_task(n) :
  M = choice([ -1, 1], p=[0.5, 0.5])
  A = np.concatenate([np.ones(n//3) * d for d in [ -1, 0, 1]])
  shuffle(A)
  V = A.copy()
  V[np.where(A == 0)] = -M
  V[np.where(A == -M)] = 0
  return M, A, V
```

```
#n must be a multiple of 3, to ensure exact repartitions of directions
from numpy.random import shuffle, choice, permutation
def perfect_comod_task(n, strength) :
    M = choice([ -1, 1], p=[0.5, 0.5])
    A = np.concatenate([np.ones(n//3) * d for d in [ -1, 0, 1]])
    shuffle(A)
    V = A.copy()
    V[np.where(A == 0)] = -M
    V[np.where(A == -M)] = 0
    r_n = int(n * (1 - strength))
    random_idxs = choice(n, r_n, replace=False)
    A[random_idxs] = A[random_idxs][permutation(r_n)]
    V[random_idxs] = V[random_idxs][permutation(r_n)]
    return M, A, V
```

### Probabilistically balanced comodulation task

```
def probabilistically_balanced_comod_trial(n, pcc, pii):
    pc = (1+pii -3*pcc)/2
    pi = (1+pcc -3*pii)/2
    return comod_trial(n, pcc, pii, 0, pc/2, pi/2, 0)
```

### Detection tasks

```
# Detection task trial
from numpy.random import choice
def detection_trial(n, pm, pe, pn, pc, pi):
    M = choice([ -1, 0, 1], p=[pm/2, 1 -pm, pm/2])
    E = []; A = []; V = []
    for t in range(n):
        # emit variable depends on M
        if M:
            e = choice([0, 1], p=[1 -pe, pe])
        else:
            e = 0
        # distribution of A and V depends on M, E
        if e:
            vals = [ -M, 0, M]
            p = [pi, 1 -pc -pi, pc]
        else:
            vals = [ -1, 0, 1]
            p = [pn/2, 1 -pn, pn/2]
        # make random choices and append
        A.append(choice(vals, p=p))
        V.append(choice(vals, p=p))
        E.append(e)
    return M, E, A, V
```

### Multichannel, multiclass detection task

```
import numpy as np
from numpy.random import choice
def trials(num_channels, num_classes, pe, pc, time_steps=90, repeats=10000):
    M = np.random.randint(num_classes, size=repeats)
    E = choice(np.arange(2), p=[1 -pe, pe], size=(repeats, time_steps))
    E[M==0, :] = 0
    # use this when E=0
    C0 = np.random.randint(num_classes, size=(repeats, time_steps, num_channels))
    # use this when E=1, initially set up so that correct answer is 0, then shift by M
    p = np.ones(num_classes)*(1 -pc)/(num_classes -1)
    p[0] = pc
    C1 = choice(np.arange(num_classes), size=(repeats, time_steps, num_channels), p=p)
    C1 = (C1+M[:, None, None]) % num_classes
    C = C1*E[:, :, None]+C0*(1 -E[:, :, None])
    return M, E, C
```

### Continuous detection task

```
import numpy as np
from numpy.random import rand, randn
def generate_trials(num_trials, num_channels, num_windows, pm, pe, mu, sigma):
    M = choice([ -1, 0, 1], size=(num_trials,), p=[pm/2, 1 -pm, pm/2])
    # We choose E values for all trials and windows, but then multiply by 0 where M=0.
    # The output has shape (num_trials, num_channels, num_windows) which we'll want below
    E = np.array(rand(num_trials, num_windows)[:, None, :]<pe*(M!=0)[:, None, None],
                 dtype=float)
    # Now we compute the mean and std for the normal distribution for each point
    mu = mu*E*M[:, None, None]
    sigma = 1 -E+sigma*E
    # Then generate normal random samples according to this
    X = randn(num_trials, num_channels, num_windows)*sigma+mu
    return M, X
```

## E. Derivations

Following equation 9 (Methods: Bayesian models) to compute the maximum a posteriori (MAP) estimate for the nonlinear fusion algorithm we simply need to compute the following for each task:

$$\log P(\mathbf{C}_t \mid M = m) \tag{24}$$

If we want to compute the MAP estimator for linear fusion we make the additional assumption that each channel is independent, i.e. assume:

$$\log P(\mathbf{C}_t \mid M = m) = \sum_{i=1}^{n} \log P(C_t^i \mid M = m) \tag{25}$$

**Classical and probabilistic comodulation tasks**

For the classical task, for example $\mathbf{C}_t = (A_t, V_t)$ where $A_t$ and $V_t$ are conditionally independent given $M$ so:

$$\log P(\mathbf{C}_t \mid M = m) = \log P(A_t \mid M = m) + \log P(V_t \mid M = m) \tag{26}$$

where

$$\log P(A_t \mid M = m) = \begin{cases} p_c & \text{if } A_t = m \\ p_i & \text{if } A_t = -m \\ p_n & \text{if } A_t = 0 \end{cases} \tag{27}$$

and similarly for $V_t$.

For the probabilistic comodulation task, $\log P(\mathbf{C}_t \mid M = m)$ is just one of the values $p_{cc}$, $p_{cn}$, etc. depending on the values of $\mathbf{C}_t$ compared to $m$. For example, $\log P(\mathbf{C}_t = (m, 0) \mid M = m) = p_{cn}$.

**Detection tasks**

For the detection tasks, we cannot observe the latent variable $E_t$ so we have to marginalise over all possible values and then use conditional independence of channels given $M$ and $E_t$:

$$\begin{aligned} \log P(\mathbf{C}_t \mid M = m) &= \log \sum_{e=0}^{1} P(\mathbf{C}_t \mid M = m, E_t = e) P(E_t = e \mid M = m) \\ &= \log \sum_{e=0}^{1} \prod_{i=1}^{N_C} P(C_t^i \mid M = m, E_t = e) P(E_t = e \mid M = m) \end{aligned} \tag{28}$$

**Multichannel, multiclass detection task**

We derive the estimator for the isotropic case by substituting equation 7 (Methods: Multichannel, multiclass detection task) into (28). To simplify this, we use the following notation:

$$\begin{aligned} p_i &= (1 - p_c)/(N_D - 1) && \text{probability of incorrect observation} \\ p_n &= 1/N_D && \text{probability of any observation when } E_t = 0 \\ x(t, m) &= \#\{i : C_t^i = m\} && \text{the number of observations equal to } m \text{ at time } t \end{aligned} \tag{29}$$

With this, noting that $P(C_t^i | M = m, E_t = 1) = p_c$ if $C_t^i = m$ or $p_i$ otherwise, and that this happens $x_t(m)$ and $N_C - x_t(m)$ times (respectively) in the product over $i$:

$$\begin{aligned} \log P(\mathbf{C}_t | M = m) &= \log(p_c^{x(t,m)} p_i^{N_C - x(t,m)} p_e + p_n^{N_C}(1 - p_e)) \\ &= \log((p_c/p_i)^{x(t,m)} p_i^{N_C} p_e + p_n^{N_C}(1 - p_e)) \\ &= \log(\alpha + \beta\gamma^{x(t,m)}) \\ &= \log(1 + b e^{c x(t,m)}) + a \end{aligned} \tag{30}$$

where

$$
\begin{aligned}
\alpha &= p_n^{N_C}(1-p_e) \\
\beta &= p_i^{N_C} p_e \\
\gamma &= p_c/p_i \\
a &= \log\alpha \\
b &= \alpha/\beta \\
c &= \log\gamma
\end{aligned} \tag{31}
$$

This is the softplus function mentioned in Results: The softplus nonlinearity solves a wide class of multimodal tasks.

In the general case of (28) where we do not assume isotropy, we need to estimate $(N_D^{N_C}-1)N_D$ parameters. The classical evidence weighting approach assumes conditional independence of channels and is equivalent to the case $P(E_t=1)=0$, giving $(N_D-1)N_C N_D$ parameters $P(C_t^i|M=m)$ as there are $N_C$ values of $i$, $N_D$ values of $m$ and for each $i$ and $m$ there are $N_D$ probabilities (but these have to sum to 1 so $N_D-1$ parameters). Allowing for $E_t=1$ gives us parameters $P(C_t^i|M=m,E_t=e)$ and $P(E_t=e|M=m)$ however when $E_t=0$ the parameter $P(C_t^i|M=m,E_t=0)$ cannot depend on $m$ by definition. We therefore have an additional $N_C(N_D-1)$ parameters from the $P(C_t^i|M=m,E_t=e)$ term and an additional $N_D$ parameters from the $P(E_t=e|M=m)$ term. The total extra number of parameters is then $N_C(N_D-1)+N_D$ or approximately a fraction $1/N_D$ of the number of parameters for evidence weighting.

**Continuous detection task**

In general, if $C_t^i$ has a continuous rather than discrete distribution, (28) still holds but this time interpreting $P(C_t^i \mid M=m, E_t=e)$ as a probability density function rather than a discrete probability.

In Results: The softplus nonlinearity solves a wide class of multimodal tasks, we show results from a special case where $C_t^i|E_t=0 \sim N(0,1)$ and $C_t^i|E_t=1 \sim N(\mu M,\sigma^2)$. In this case:

$$
\begin{aligned}
P(C_t^i=c_t^i|M=m,E_t=0) &= \phi(0,1,c_t^i) \\
P(C_t^i=c_t^i|M=m,E_t=1) &= \phi(\mu m,\sigma,c_t^i) \\
\phi(\mu,\sigma,c) &= \frac{1}{\sqrt{2\pi}\sigma}\exp -(c-\mu)^2/2\sigma^2
\end{aligned} \tag{32}
$$

Expanding this out gives:

$$
\begin{aligned}
\log P(\mathbf{C}_t=\mathbf{c}_t|M=m) &= \log\left((1-p_e)\prod_i \phi(0,1,c_t^i)+p_e\prod_i\phi(\mu m,\sigma,c_t^i)\right) \\
&= \left(\log\frac{1-p_e}{(2\pi)^{N_C/2}\sigma^{N_C}}\right)\sum_i (c_t^i)^2 + \\
&\quad \log\left(1+\frac{p_e}{1-p_e}\exp\left\{\frac{1}{2\sigma^2}\sum_i(c_t^i-\mu m)^2-\frac{1}{2}\sum_i(c_t^i)^2\right\}\right) \\
&= (\text{term with no } m) + \text{softplus}(\text{quadratic}(m,\mathbf{c}_t))
\end{aligned} \tag{33}
$$

## References

[1] Denis P. Probabilistic inference using generators - the statues algorithm. arXiv. 2018. doi: 10.48550/ARXIV.1806.09997.
