# Supplementary material for: Nonlinear fusion is optimal for a wide class of multisensory tasks
Source: PLoS Comput Biol. 2024 Jul 5;20(7):e1012246. doi: 10.1371/journal.pcbi.1012246 (PMC11253934; doi:10.1371/journal.pcbi.1012246)
Supplement: Attachment — Submitted filename: Ghosh_2024_responses_to_reviewers.pdf [file pcbi.1012246.s002.pdf]

To whom it may concern,

Thank you for considering our manuscript and for taking the time to provide such clear and well thought out suggestions.

Broadly speaking, all three reviewers agreed with our methodology and results. Reviewer 1 stated that our "analyses are thoughtful, and the methodology is sound", and similarly reviewer 3 wrote that "the paper is well written and technically impressive, with elegant analyses".

That said, all reviewers highlighted that our work would benefit from an alternative framing, that more appropriately placed it in the context of prior work.

With that in mind, we have reframed our manuscript as comparing linear vs nonlinear fusion, as opposed to the accumulate-then-fuse and fuse-then-accumulate terminology which we introduced in our original submission. Based on this, we have re-titled our manuscript, re-written the abstract and introduction and made revisions throughout.

Below, we include the original reviews, divided into actionable items. For almost all items we have updated our manuscript in line with the reviewers' suggestions, though when appropriate we have simply replied directly. For both cases we include our replies (in italics) below.

We are confident that these changes have strengthened our manuscript and look forward to hearing from you.

Reviewer 1

In the present study, the authors investigated two conceptual frameworks for multisensory decision making, fuse-then-accumulate (FtA) and accumulate-then-fuse (AtF), on a series of novel multimodal tasks. By design, these "comodulation" tasks entail coincidence detection between channels, and thus require the "multimodal" unit in FtA, as shown by an excelled performance of trained spiking neural networks with FtA on the comodulation tasks, but not on the classical tasks. Combining simulation with an ideal observer model, the authors provided a deeper understanding of why the nonlinearity embedded in the FtA framework is important and how the signal sparsity plays a role. Finally, the authors explored the link between single neuron properties and the super-additive nonlinearity in FtA. Together, the authors argue that the FtA framework is optimal for a wide range of multisensory problems and may be widely adopted by biological systems.

The manuscript is overall well-organized, the analyses are thoughtful, and the methodology is sound. The discussions of the series of comodulation tasks are also insightful. However, I have several major issues, mainly related to interpreting the results and placing the claims properly in the context of previous literature, along with other minor comments.

Major

1. FtA is not new.

i) In fact, the large body of early multisensory studies, such as those from Barry Stein and colleagues, all point to the FtA framework. For instance, the superadditivity and inverse effectiveness of multimodal units in SC have been suggested to facilitate near-threshold (i.e., "sparse") signal detection (see, e.g., Stein and Stanford, 2008, https://doi.org/10.1038/nrn2331), very similar to what the authors propose here.

Hopefully our contribution, a detailed comparison of linear and nonlinear fusion across a wide class of multisensory tasks, is clearer from our reframing of the paper. Additionally, we have limited our use of novel/new to describing the new tasks which we introduce.

ii) Even Fetsch et al, 2013 and the series of work from Angelaki and DeAngelis did not suggest an AtF algorithm (Line 46). Although they did not report a superadditivity in cortical areas, their working hypothesis was still FtA, in the sense that visual and vestibular self-motion cues are first fused in multisensory areas such as MST and then accumulated over time in decision making areas such as LIP. (But see a follow-up study suggesting "accumulate-while-fuse" or "late-convergence" in the same system: Hou et al., 2019, https://doi.org/10.1016/j.neuron.2019.08.038). iii) Similarly, I don't think Coen et al., 2023 has suggested AtF either (Line 50). It is clearly proposed in their Figure 7 that MOs first fuses visual and auditory cues, and then a downstream integrator accumulates MOs activity, a framework consistent with FtA. iv) The debate between FtA and AtF is also not new, which should not be overlooked in this study. See for example Bizley et al., 2016, https://doi.org/10.1016/j.conb.2016.06.003. Together, it is inappropriate to claim that the authors "propose a new FtA algorithm" (line 65). Rather, the present study provides, at most, detailed analyses of tasks where coincidence detection of multimodal signals is important and where fusion before accumulation is required. The novelty is not the FtA framework per se, but the generalization of "coincidence detection" from near-threshold stimuli in terms of signal amplitude to those in terms of temporal sparsity. The manuscript, especially the Abstract, Introduction, and Discussion, should be rewritten accordingly.

We agree that our previous terminology (AtF and FtA) was imprecise as it emphasised early vs late fusion (as in Bizley et al., 2016) rather than linear vs nonlinear fusion (as intended). However, if all the operations in a network/model are linear then the order does not matter, and early and late fusion would be equivalent. Consequently, we group these models under a common "linear fusion" term. We believe this is an important point to make and so have added text to our introduction [line 49] and a mathematical explanation to our supplementary material [line 630].

2. Taking a step back, the dichotomy between FtA and AtF may itself be debatable, especially in the context of distributed computation across interconnected brain regions. From early sensory processing to perceptual decision making, multiple processes of "fusion" and "accumulation" could occur at different hierarchies and on different timescales. In this case, how can one define the order of their occurrence? In addition, as suggested in Hou et al., 2019 and Coen et al., 2023, they could also happen simultaneously in the same neural circuit. The authors should therefore discuss the limitations of the oversimplified network architectures in Figure 1, and provide a stronger link to biological reality by maybe suggesting where experimentalists should test the predictions of the present study in the brain.

We agree that our work uses a simplified network architecture and have added this limitation to our discussion as suggested [line 315]: "we focused on small network models with simple architectures. This is problematic as it elides the complexity of real multimodal circuits, like the presence of skip connections (Ohyama et al., 2015), and may make our results difficult to relate to real circuits. However, our multimodal architecture is optimal for all the tasks we considered - suggesting that additional complexity may only be necessary for even more challenging tasks, and the multimodal units in our model simply represent the first neurons to receive inputs from multiple modalities - wherever they may be found in the brain."

3. The connection between Section 3.5.2 and the rest of the paper is not very clear.

I) How does the inverse effectiveness of single cells contribute to the nonlinearity needed to solve the task? What is the relationship between single cell nonlinearity and network nonlinearity? Why do we need both?

To solve our tasks observers must assign coincidence a higher weight than the sum of the individual observations from each channel. As such, multimodal units must use nonlinear or super-additive activation functions, and networks which include these units can behave nonlinearly. In contrast, networks with linear units can only implement linear functions. We have added an intuitive explanation of this to the end of section 3.2 [line 125]. Furthermore "As neurons naturally transform their inputs via a spiking nonlinearity, and we show that the exact form of this nonlinearity is not critical" we argue that "nonlinear fusion constitutes a more natural solution for networks of spiking neurons." [line 329].

II) In particular, in Figure 4C, why is the superadditivity of the Cl and Dd tasks even larger than that of the Cm+ task?

In general, it is difficult to interpret how single neuron properties link to network behaviour through single unit correlations as in Figure 4C. This is what motivates our more thorough causal analysis in Figures 4D, S5 and S6. In this specific case, while we observe slightly lower mean additivity in the Cm+ task, as for the Ds task the most additive units within each network are the most causally important for the Cm+ task.

III) In Section 3.5.2 and Figure 4B, single neurons with shorter tau were associated with higher additivity values, whereas in Figure 8.S6, additivity is positively correlated with tau in the Cm+ task. Why is this?

It is interesting that this relation seems inverted here, but this is not mathematically inconsistent. Tau is negatively correlated with additivity across the full population of possible neural parameters, but this correlation doesn't have to be the case for the subset of neural parameters that were found to be optimal for a particular task (in this case the Cm+ task). For example, in the figure below the black and red dots together are positively correlated, but the red dots coming from a subset of the distribution are negatively correlated. So, as above, correlative results are difficult to interpret, and our causal results are consistent between the Ds and Cm+ task.

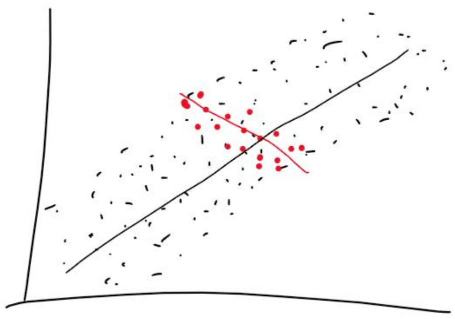

Minor

1. Figure 1.C2: in the probabilistic version of the comodulation task, what is the correct answer for a trial where L > R but LL < RR?

For each trial the correct answer is defined by the hidden variable M (the target motion to be inferred).

2. Line 132: what do you mean by ceiling accuracy? How could the performance better than it?

We have reworded this phrase for clarity [line 144]: "To meaningfully compare the two algorithms we then filtered these results by discarding parameter sets which were either trivially easy or overly difficult", and have added a methods section (5.6.1, line 510) which details how we implement this.

3. Line 144: how does "sparsity of the signal" depend on p_e and p_c? Is it "sparsity of the correct signal"?

We agree this was unclear. We have reworded it as "the sparsity of the useful signal buried in the noise". The sparsity on the axis of the figure is essentially just p_c varying from about 0.35 to 0.95, with p_e varying from about 0.27 (when $p_c \approx 0.35$ in the dense case) to 0.07 (when $p_c \approx 0.95$ in the sparse case). This might be clearer with the full plot of the variation of the accuracy of the fuse-then-accumulate (nonlinear) algorithm with p_c and p_e:

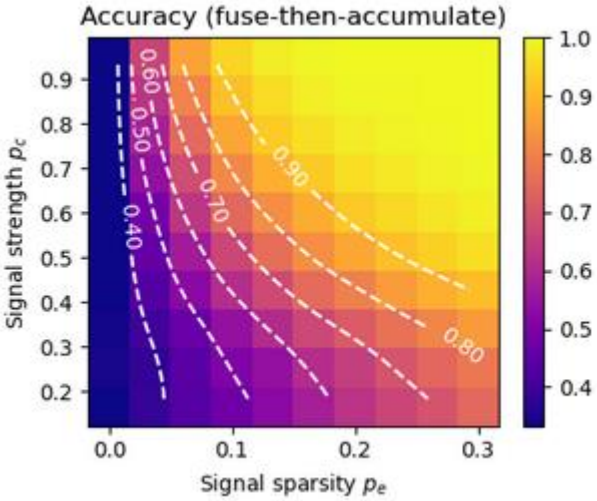

The point is that p_c and p_e must be jointly manipulated to get a fixed accuracy of 80% (moving along the 0.80 contour in the plot above), and accuracy is monotonic in both p_c and p_e and both could reasonably be described as giving a "sparsity" of useful information which is why we didn't want to identify just one of these variables as the axis. We did check if it was simply the product $p_c p_e$ that determined accuracy (this would make sense intuitively) but it wasn't as simple as that unfortunately. The exact calculation is described in the methods section "Detection task" (last paragraph).

4. Equation (1): please provide some intuition as to why nonlinearity is important for solving a sparse, coincidence-detector-like task.

The basic idea can be understood with a simple example of two observations. Let's say in situation 1, at time t=0 you observe (Audio=Left, Video=Neutral) and at time t=1 you observe (A=N, V=L). In situation 2, you observe (A=L, V=L) and (A=N, V=N). For a linear model, both these situations must provide exactly the same amount of evidence in favour of L because each modality and each time step are independent (and equally reliable by assumption), and there are the same number of left

observations. However, a nonlinear model can give more weight to the observation (A=L, V=L) at a single time step t=0 in situation 2. We have added this to the end of the last paragraph of the results section "Multimodal units are critical for extracting comodulated information" [line 125] and we think this is a better place to explain this than equation 1, which is about the more complicated case with multiple channels and directions (though the same intuition holds).

5. Section 3.5.2: method Section 5.4.1 should be mentioned here.

We have updated the text to include this reference.

6. Figure 5: each trial's alpha (transparency?) is not distinguishable.

In this figure, the alpha (transparency) value shows how consistently 10 SNN models (for each architecture) made the same decision (L/N/R). As networks tended to make the same decisions, most of these values are simply 1. Except for the panel which we highlight in the figure legend. We have added text to the legend to make this clearer: "Otherwise networks choose consistently - so most trials have high alpha.". We also experimented with alternative colours and methods for setting alpha, though concluded that our approach best represented our data.

Reviewer 2

Overall, I think this manuscript makes a valuable contribution to the multisensory research field and will be of wider interest to sensory scientists interested in how cues are fused. The papers strength is in tackling a problem at 4 different levels, each of which supports and extends the conclusions of the other and at the end generates testable biological hypothesis. By implementing minimal artificial neural networks, Bayesian ideal observer models, spiking neurons and spiking neural networks on an identical family of "multisensory" tasks the authors are able to demonstrate a specific set of stimulus conditions in which it is advantageous to fuse information across "senses" before accumulating information. By exploring networks of spiking neurons and ablating targeted populations of cells they are able to demonstrate that in stimulus conditions in which fusing before accumulating (FtA) was advantageous model performance was dependent on neurons that integrated information over short timescales (i.e. coincidence detectors).

While the work is certainly interesting, and appears performed to a high standard, there are a number of places where some additional clarification would be helpful (especially for biologists; the paper is very much written for computational experts at the moment). Generally, I found the manuscript to be posed at quite a high level; probably a computational expert it is clear, but as a biologist I found several places (e.g. section 3.4) where some additional explanations would have been really helpful.

A paragraph at the beginning of the results that provided a big picture roadmap of the approach to be taken and what would be learned by each would provide a framework in which to hang the subsequent results.

Agreed. We have added a paragraph to the end of our introduction which fulfils this role [line 66].

I also found it quite confusing to go from networks, to algorithms and then back to networks; terminology is not always clear. For example, how are the 'multimodal' model architectures are different from the 'fuse then accumulate' – they seem like the same model from figure 1 and the text in sections 3.1 and 3.2 doesn't differentiate them, but I'm pretty sure the later is an ideal observer model (as in section 3.3) and the former an artificial neural network? Or do these not relate to the original networks developed for Figure 1?

We have checked our manuscript to ensure that our use of network, model, algorithm etc is as consistent as possible. As you say, Figure 1$A_{1\text{-}3}$ show network architectures while Figure 1D and E show schematics of ideal observer algorithms. The two are related as our approach to "understanding" network computations is to think of them as approximating ideal algorithms.

Figure 1C & 2F,G – what is time here? The time to a decision? The timebin over which integration is performed? What are the units?

For all our tasks we use trials with 90-time steps, where at each step we present left/neutral/right signals per channel. In these figures time denotes the number of elapsed time steps within a trial. For clarity we have added this definition to all figures which include this axis.

In the comodulation task it seems obvious that to solve the task there must be some element that looks across both channels with sufficient temporal resolution to pick up coherent signals; by definition (unless accumulation is very short-term) this is not going to be possible with a AtF model (as evidenced by chance level performance in the comodulation task (Fig.1C2)). Its not clear to me how this problem is overcome by linear integration when the signals are denser; can the authors provide some intuition as to what it is in the signal domain that changes? Or is the direction not signalled via comodulation here? I think the lines "E" in whether the signal is emitting or not but its not obvious how an observer knows what is signal vs noise (unless its via comodulation, but then why does E=1 cover the comodulated and unisensory events?). Is it simply that due to the probabilistic nature of the stimulus generation there genuinely is slightly more signal in the comodulated direction than the null direction and that making the signal denser highlights this difference? Or am I missing something fundamental…?

As you highlight, our comodulation tasks are unsolvable via linear fusion (previously termed AtF) as there is no bias in either direction (L==R), and information is only carried by coincidences across channels (LL > RR). In contrast, in our detection task, where we explore the effect of signal density, there is information from both bias and coincidence. In the dense case, the bias is large, so the task is solvable by linearly summing the information (across time and channels). Notably, E is hidden from the observer, so this sum must involve the whole trial (both when E=0 and E=1). In the sparse case, there is some bias in the correct direction, so linear fusion performs above chance (Figure 2G, purple lines). However, coincidences are informative (of the true direction) and so nonlinear fusion improves performance (Figure 2G, green and grey lines).

Relatedly, have you explored temporal windows of integration in the earlier models? Fusing and then accumulating allows a network to work on both short and longer timescales (as the network analysis later in the paper points out, and which is required if you want to both remember the past and detect coincident events across channels). Presumably at some point if evidence accumulation is sufficiently short-term it effectively becomes coincidence detection and therefore both the timescale over which the stimuli unfold and the timescale over which an evidence accumulation centre accumulates unisensory information together dictate to what extent there is a benefit to a prior fusion stage? Is there any way of exploring this parameter to relate it to the time constants within the spiking neural networks?

This is an interesting point, and we are currently exploring tasks with more temporal structure and models which can leverage that structure (ideal observer models with temporal windows of integration, and recurrent neural networks). However, this work is enough of an extension to merit a follow-up paper.

In figure 5 it seems like the really interesting trials are those highlighted by the arrows in the dense detection task. What is it about those trials that result in opposing predictions; are they trials with particularly high/low numbers of comodulated signal intervals or anything interpretable like that? Could a psychophysicists probe these trials specifically to understand the strategies of their observers?

Thank you for highlighting this. "These trials are essentially linear-nonlinear conflict trials as there is an overall bias in one direction (e.g. L > R) but more coincident information in the other (e.g. LL < RR)." which could indeed by tested experimentally. We have added the preceding text (in quotation marks) to the paper [line 278].

There are some really trivial changes that would ease the cognitive burden when reading the manuscript – for example on figure 5, why not label the columns Classic / Sparse / Dense / Comodulated? On the delta accuracy plots, specify that this is a difference in % correct etc.

We have updated the subplot headings as suggested.

The discussion could make it clearer that these results relates to a very specific subset of multisensory decision making tasks that require evidence accumulation rather than other temporally restricted, but common, multisensory tasks (such as sensory illusions, lip reading etc).

We have added the following sentence to the limitation section in our discussion: "In a similar vein, extending our work to consider non-evidence accumulation-based tasks would further generalise our results." [line 314].

While I agree that most experimental studies of evidence accumulation have focussed in the idea or indeed found evidence in favour of accumulating within a modality and then fusing across modalities, it (1) feels a little inaccurate to say this is a canonical perspective (line 44) for all multisensory tasks when there is now very good evidence that there aren't really 'unisensory' areas in cortex and (2) it feels a little disingenuous to try and claim that this is the first suggestion of a FtA perspective (line 65). This is certainly the first rigorous computational exploration of the two possibilities, but I think its far from the first study to suggest multisensory information might be integrated prior to a decision-making step.

Hopefully our refocus on linear vs nonlinear integration makes our contribution clearer. Additionally, rather than presenting linear fusion as the "canonical perspective" we now make clear the body of work which supports it [line 54].

Line 101 – I think this should be trial's rather than trials?

Thank you for the correction.

<u>Reviewer 3</u>

Ghose et al. present an interesting ML-flavored treatment of multisensory integration and coincidence detection in spiking networks. The approach is fairly novel and should be quite useful for understanding certain kinds of information integration in the nervous system. I especially like the connection to prey capture, and the generalization to cases where Nc channels are used to discriminate Nd classes.

I'm less convinced (1) that they have provided the best/only explanation for *why* multimodal units exist in the brain (though the question is an important one, given the logic they lay out about linearity and the decision stage). I also (2) disagree that the 'classical' paradigm fails to apply outside of the

laboratory, or that comodulation/coincidence detection is more naturalistic; indeed it may be implausible without some modifications to account for the temporal mismatch across neural representations for most modality pairings. Lastly, (3) I think their exposition at the outset — particularly the dichotomies of AtF vs. FtA and classical vs. comodulated — is incomplete at best, and may be perplexing or alienating to a significant portion of the field. I'll explain what I mean below. Otherwise, the paper is well written and technically impressive, with elegant analyses and nice figures, so I'm rather positive about the contribution.

I'll tackle the above three general comments in reverse order:

(3) First I think the setup and assumptions here may reflect a misreading of the literature. Arguably the 'classic' approach dates back to Landy, Maloney, Jacobs and others in the 90's, with its most cited exemplar being Ernst & Banks 2002. Notably there was no accumulation whatsoever in these models, as they only considered one sample per channel per trial. Of course many earlier papers considered temporal effects in multisensory integration, but mostly for detection or simple reaction time (Raab 1962), and few had anything resembling an accumulator model.

So when considering integration with a sequence of samples in each channel, the canonical view would have to be something like the model of Drugowitsch et al. 2014 (though one might see it foreshadowed in the probabilistic population code papers of Ma et al. 2006 and Beck et al. 2008.) In this framework, unisensory neurons represent the 'momentary evidence' in a given channel at each time step, and multisensory neurons combine this momentary evidence in a reliability-weighted fashion; again with no significant temporal accumulation. Only then is the combined evidence accumulated over time (by a downstream decision network) until a threshold is reached.

Prior to reading the current manuscript, this is what I would call fuse-then-accumulate, or what Jenny Bizley (Bizley, Jones, & Town 2016) and Yong Gu (Liu, Shan, & Gu 2023) have termed 'early' integration. So it came as a surprise to hear that most authors (including myself :) consider accumulate-then-fuse to be the standard model. [But if I'm wrong and, say, Trommershauser et al. explicitly endorse AtF, then let me know!]

Interestingly, there is more recent evidence from Gu and others that decision-related neurons appear to accumulate evidence arriving separately from unisensory channels, rather than from a common multisensory representation. This could be called 'late' integration, or perhaps accumulate-AND-fuse-at-the-same-time. Lastly, in this taxonomy, accumulate-THEN-fuse would be a kind of race model: separate bounded accumulators for the two modalities, each of which reaches its own 'decision', and the behavioral choice is selected from the two decisions by a (weighted) coin flip. This sort of mechanism is ruled out by reaction time data in studies like Drugowitsch et al. 2014, but might be at play in other tasks; see also Jerjian et al., 2023. (Note the distinction between the latter two models depends on where the termination rule is applied; this is perhaps why the current paper's framework, which lacks a termination rule, fails to make the distinction.)

Anyway, you get the picture. The point is that AtF as defined here is hardly canonical or classical, even if it appears that way from Coen et al. 2023. I'd argue FtA is actually closer to what many folks consider standard, though generally without an explicit nonlinearity (but not ruling one out either), and not typically capable of precise coincidence detection. These latter features, and the associated derivations and network models, make the current work novel and exciting, well worth sharing. But in order to situate the theory better within the current literature I think a revised intro/discussion are needed which cover the above considerations—and either clarify why AtF should be considered canonical/optimal, or avoid assigning it those labels.

We agree that our previous terminology (AtF and FtA) was imprecise as it emphasised early vs late fusion (as in Bizley et al., 2016) rather than linear vs nonlinear fusion (as intended). As suggested, we have reframed our manuscript by re-titling it, rewriting our abstract and introduction and making revisions throughout.

(2) Secondly, although the distinction between ‘classical’ and ‘comodulation’ is an interesting one, I don’t see how it calls into question the relevance of the classical scenario outside of a laboratory context. Surely using two streams of evidence to improve the inference of their common cause is broadly relevant, even with the conditional independence and linearity assumptions. Whether the decision rule explicitly looks for comodulation on a fine time scale for a given task is an empirical question, not a redefinition of which class of task is ecologically valid.

We have removed our question on “relevance outside of a laboratory context” from the introduction.

Plus, for many cue pairings, strict comodulation is implausible anyway, because the dynamics of stimulus encoding destroy any such correlations in the input. Example 1: the visual motion system encodes velocity, whereas vestibular otolith signals follow acceleration. Example 2: tactile texture is encoded in dynamic spiking patterns linked with motion of the object over the skin, whereas visual texture is represented in a rate code through rapid integration of higher-order statistics. Even simple latency differences (e.g. auditory<visual)>

Arguably the introduction of appropriate delays will compensate for latency mismatches between senses. In our model, for example, if signals arrive from channel 1 one-time step later than those from channel 0 (picture applying this offset to Figure 1B2) we can simply delay the signals from channel 0. A related (in preparation) project shows that neural networks can co-learn their weights and these delays, and this seems biologically plausible too. In the auditory system, for example, sounds arrive at each ear at a different time, but axonal delays make sound localisation possible. Based on a later comment we have added a sentence on this: “Notably, in natural settings signals from separate channels will likely arrive and be processed at different speeds. However, it is reasonable to assume that these temporal mismatches can be compensated for by appropriate delays.” [line 164].

On the other hand, we agree that strict comodulation seems unrealistic and explain this in our discussion: “both the classical and comodulation tasks seem unrealistic for two reasons. First, both are composed of dense signals (i.e. every time step is informative). Second, both treat the relations between channels in extreme ways: in the former, the temporal structure of the joint multimodal signal carries no information, while in the latter all the information is carried in the joint signal and the task is impossible using only one modality.” [line 292].

(1) Thirdly, the question of why we even have multimodal units is not as untouched by previous work as the authors imply. For instance, in most models (including Drugowitsch et al.) these units are the site of the reliability-weighting step. We don’t know the exact mechanism, but it could very well involve nonlinearities, and thus provide a rationale for the kinds of computations shown in the current paper but without invoking coincidence detection at all.

It is true that Drugowitsch et al. (2014) discuss nonlinear operations in the reliability-weighting step, but these are not the nonlinearities we focus on, observed in for example Stanford et al. (2005), where a combined audio+video signal gives a stronger response than the sum of just audio or just video (i.e. super-additivity). We have reworded the introduction (fourth paragraph) to make this clearer [line 62].

[On that note, it was unclear which of the author's models achieves reliability-based cue weighting; perhaps it is trivial with the linear operation at the decision unit but it would be reassuring to see a simulation of this, ie the cue-conflict conditions.

Reliability-based weighting makes sense for linear models (in our sense of being linear in combinations of senses) and when channels are conditionally independent (given a target variable), which is not the case for the new tasks we consider. When channels have a joint distribution, we cannot linearly weight them for reliability. Instead, different channel reliabilities, in our detection task, could be modelled by dropping the assumption $P(A_t = M|E_t = 1, M) = P(V_t = M|E_t = 1, M)$ *(equation 6 in section "Detection task" in methods), which will change the log-odds of pairs of observations* $log\,P(C_t|M = m)$ *in equation 9 (section "Bayesian models" of methods). Computationally, this can be included in various combinations of the weights in the artificial and spiking neural networks (but cannot only be included in the linear readout since it changes the joint distribution). More generally, when channels are not conditionally independent, reliability of channels doesn't really apply as the joint distribution can depend on the actual values observed in the different channels in arbitrarily complicated ways that cannot be accounted for by a linear weighting of channels. This seemed like an interesting and not at all obvious observation and therefore we have mentioned it in the Discussion [line 320] and a new section of the Supplementary information [line 634].*

And why does accuracy go down on conflict trials (Fig 7)?]

In the "extended classical task" (Figure S1) each channel has a 'true' direction and each channel has a 'strength', and we label the overall true direction to be the true direction of the channel with the higher strength. However, which channel has the higher strength is unknown to the model, so including a second channel giving conflicting information (conflict trials) adds more noise than having no information from the second channel (unimodal trials).

Lastly, although causal inference was mentioned briefly, other more recent papers are perhaps worth citing because they give further rationale for multimodal neurons, specifically 'congruent' and 'opposite' neurons which may implement causal inference (concurrent segregation and integration): Rideaux et al. PNAS 2021, and Zhang et al. Elife 2019.

We have added these references to our discussion.

Minor/specific:

56: The fact that behavior and neurons in untrained mice do not show linear summation / AtF is an issue with the Coen et al. paradigm and/or the brain area they examine, not a general problem for the algorithm (even though, per above, I don't think the version of AtF described here is really the default/classic view).

We have removed this point re Coen et al., 2023.

85-87: I can see how the different architectures described here accumulate evidence at similar rates, but I'm not sure this resolves the speed-accuracy tradeoff (SAT) question. For SAT you need a termination rule (ie bound), and once you have one, discrepancies may appear when including different levels of reliability (signal to noise) in the two channels. This is how Drugowitsch was able to rule out two kinds of race model: one, which I associate with AtF (see above), predicts that RT on multimodal trials would be yoked to the slowest of the two accumulators (the low-reliability channel), whereas empirically it is intermediate between them and often nearly as fast as the high-reliability channel. [And if the idea is that the termination rule is only applied to the 'fuse' stage of AtF, this is

equivalent to “accumulate-AND-fuse” aka late integration. In other words, without a termination rule there is no functional role for accumulation, so the accumulate units in AtF seem pointless.] The other version of the race model is where the races remain independent and the first modality to reach its bound governs the choice and RT. This predicts RTs faster than empirically observed, and cannot explain improvement in psychophysical thresholds on combined vs. unisensory trials.

—> Sorry I don’t have a TL;DR suggestion here; I guess I’m questioning the relevance of Fig. 1.C1 in the point being made about “what computational role do these units play.”

In Figure 1C1 all architectures accumulate evidence at the optimal rate (shown by the ideal nonlinear fusion curve in grey). In contrast, in Figure 2G, all architectures accumulate evidence, but do so at different rates. Hopefully this makes it clear that our setup can detect differences in speed/accuracy between observers.

136-150: This part is cool. Totally on board here. Might be a good place to bring in ways to handle the temporal mismatch issue (point (2) above), if possible.

Thank you. As suggested, we have added a sentence on this here: “Notably, in natural settings signals from separate channels will likely arrive and be processed at different speeds. However, it is reasonable to assume that these temporal mismatches can be compensated for by appropriate delays.” [line 164].

Fig. 5: It wasn’t entirely clear to me how the points in the off-diagonal quadrants can tell you which algorithm is being used. Wouldn’t that require prior knowledge of the network architecture supporting the behavior (e.g. 2-unimodal vs. multimodal vs. something else not tested here)? The informativeness of the data points indicated by the arrows seems to require 2-Unimodal, for instance. This is fine for models but much more of a challenge in real brains.

In this figure, each point shows the amount of right minus left evidence each ideal Bayesian algorithm (linear fusion - x, nonlinear fusion - y) would assign to a single trial. We can colour these points by the choice (left, right, absent) any observer makes. Here, the observers are models, but they could equally be experimental subjects (mice, primates, humans etc). When examining an observer’s choices, their decision boundary indicates which algorithm their behaviour more closely resembles. A linear strategy (e.g. 2-unimodal) will be vertical. While a nonlinear strategy (e.g. multimodal) will be horizontal and other (real) observers may fall somewhere in-between.

Consequently, the marked points (in the off-diagonal quadrants) are particularly informative as the linear and nonlinear fusion algorithms predict that observers will make different choices. We have added a sentence on this: “These trials are essentially linear-nonlinear conflict trials as there is an overall bias in one direction (e.g. L > R) but more coincident information in the other (e.g. LL < RR). [line 278].

292 & 311 both contain the identical phrase “may constitute a better description of multimodal processing,” in case that was unintentional.

Thank you for noting this. We have rephrased one of these uses.

Do you want your identity to be public for this peer review? For information about this choice, including consent withdrawal, please see our Privacy Policy.

Reviewer #1: **Yes:** Han Hou

Reviewer #2: No

Reviewer #3: No
